# Cyld restrains the hyperactivation of synovial fibroblasts in inflammatory arthritis by regulating the TAK1/IKK2 signaling axis

**DOI:** 10.1038/s41419-024-06966-2

**Published:** 2024-08-09

**Authors:** Vagelis Rinotas, Kalliopi Iliaki, Lydia Pavlidi, Theodore Meletakos, George Mosialos, Marietta Armaka

**Affiliations:** 1 Institute for Fundamental Biomedical Research, Biomedical Sciences Research Center (BSRC) “Alexander Fleming”, Vari, Greece; 2 School of Biology, Aristotle University of Thessaloniki, Thessaloniki, Macedonia Greece

**Keywords:** Chronic inflammation, Mechanisms of disease, Cell signalling

## Abstract

TNF is a potent cytokine known for its involvement in physiology and pathology. In Rheumatoid Arthritis (RA), persistent TNF signals cause aberrant activation of synovial fibroblasts (SFs), the resident cells crucially involved in the inflammatory and destructive responses of the affected synovial membrane. However, the molecular switches that control the pathogenic activation of SFs remain poorly defined. Cyld is a major component of deubiquitination (DUB) machinery regulating the signaling responses towards survival/inflammation and programmed necrosis that induced by cytokines, growth factors and microbial products. Herein, we follow functional genetic approaches to understand how Cyld affects arthritogenic TNF signaling in SFs. We demonstrate that in spontaneous and induced RA models, SF-Cyld DUB deficiency deteriorates arthritic phenotypes due to increased levels of chemokines, adhesion receptors and bone-degrading enzymes generated by mutant SFs. Mechanistically, Cyld serves to restrict the TNF-induced hyperactivation of SFs by limiting Tak1-mediated signaling, and, therefore, leading to supervised NF-κB and JNK activity. However, Cyld is not critically involved in the regulation of TNF-induced death of SFs. Our results identify SF-Cyld as a regulator of TNF-mediated arthritis and inform the signaling landscape underpinning the SF responses.

## Introduction

Rheumatoid Arthritis (RA) is a complex inflammatory disease irreversibly affecting synovial joints. The thin synovial membrane is inflamed, gradually reverted into a hyperplastic and invasive tissue mass namely pannus, which causes joint destruction. The synovial fibroblasts (SFs), a major mesenchymal cell type populating synovium, actively participate in the pannus by exhibiting a proinflammatory profile with migratory and tissue-invading properties leading the tissue destruction [1].

We have previously shown that SFs exclusively receive the pathogenic load of TNF to orchestrate the arthritic disease in modeled arthritis, such as the *hTNFTg* and *TNF*^*ΔARE*^ models or Collagen Antibody Induced Arthritis (CAIA) [2, 3]. Transcriptomic and epigenomic analysis of the *hTNFTg* SFs highlighted NF-κB as a major component of inflammatory arthritic process [4]. In concert with this finding, the SF-specific IKK2 targeting in the *hTNFTg* mouse uncoupled the function of IKK2 in both TNF-induced NF-κB and death responses in vivo, highlighting the complexity of signaling events under chronic inflammatory conditions [2]. Interestingly, accumulating evidence indicates several molecular checkpoints that regulate the formation of Complexes I and II in the TNF signaling pathway leading towards survival/inflammatory signals or cell death respectively [5].

Ubiquitination is a posttranslational modification that is implicated in the regulation of signaling pathways by tagging ubiquitin (Ub) via its C terminus to a target protein directing it either for degradation or becoming scaffolds. Ub chains is formed by constitutive attachment of Ub to either of seven different lysine (K) residues (K6, K11, K27, K29, K33, K48, K63) or the N-terminal methionine (M1, Met1, linear chains) [6]. Remodeling of Ub loads of proteins is performed by specific Ub ligases or deubiquitinases (DUBs). The most well-studied types of Ub chains are the K48, which predispose to proteosomal degradation, the K63, which affects activation status of proteins, and the M1 which mainly contributes to scaffolding properties of proteins [7]. The Ub-editing enzymes A20 (encoded by *Tnfaip3*), Otulin and Cylindromatosis (Cyld) are critical components with DUB activity modifying cytokine- and growth factor-induced signaling pathways [8]. Multiple studies causally linked mutations in A20 with several inflammatory conditions, including arthritic diseases [9]. More recently, it was shown that mutations of Otulin, a DUB that removes M1 Ub chains, are associated to an autoinflammatory syndrome in humans and lethal poly-inflammatory consequences in mice [10–14]. CYLD has been originally described as a tumor suppressor gene mutated in familial cylindromatosis, predisposing to the development of cancerous lesions in the skin [15–17]. The majority of detected CYLD mutations affect either the DUB activity or its expression levels [18]. Remarkably, genome-wide association studies additionally implicated CYLD polymorphisms in inflammatory diseases [19]. Functional genetic approaches revealed that mutated Cyld is associated not only to cancerous conditions but also to immune homeostasis and inflammation [8, 20]. By removing K63 and M1 Ub- chains, CYLD regulates several signaling pathways [16, 17, 21] acting downstream of TNF, TGFbeta, Wnt/β-catenin, Hippo, Notch signals such as c-Jun N-terminal kinase (JNK), p38 mitogen-activated protein kinase (p38 MAPK)), nuclear factor-кB (NF-κB) [22–26]. Recently, CYLD had been also exhibited to function as another checkpoint that prevent RIPK1-dependent programmed necrosis induced by TNF [27].

Accumulating evidence strongly suggests that the output of CYLD is stimuli- and cell-context dependent. Even in the absence of mutations or polymorphisms, downregulation of CYLD has been detected in affected tissues of debilitating diseases such as NASH [28] or inflammatory arthritis [29]. In arthritic synovium, low levels of CYLD are inversely correlated with the NF-κB activity in RASFs [29]. However, mechanistic studies on the effects of SF-specific CYLD knockdown or loss of DUB activity in vivo remains underexplored. In this study, we pursued το decouple the CYLD involvement in the inflammatory behavior of SFs, by employing both acute and chronic TNF-dependent modeled arthritis, coupled with genetic targeting and biochemical studies on DUB-deficient Cyld SFs.

## Results

### TNF-mediated arthritis is deteriorated by mesenchymal Cyld-DUB deficiency

**Fig. 1:**
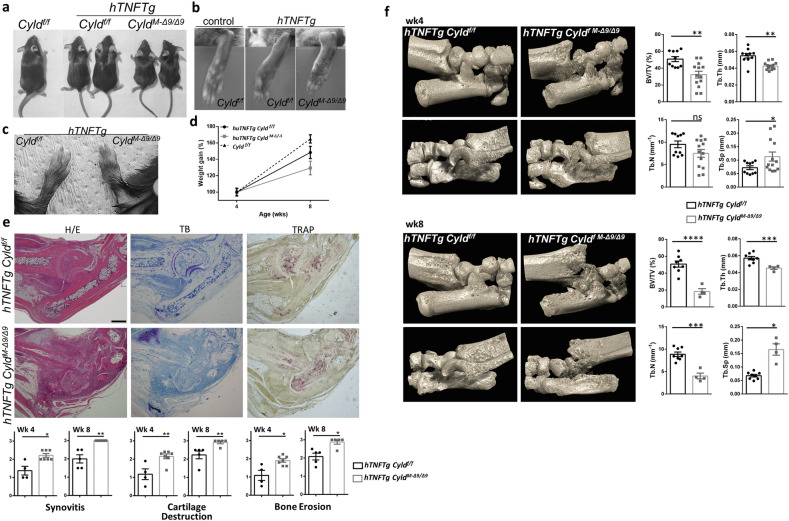
Mesenchymal Cyld-DUB deficiency deteriorates arthritic disease of *hTNFTg* mouse model. **a** Gross appearance and the corresponding **b** hindpaws and **c** forepaws of *Cyld*^*f/f*^, *hTNFTg Cyld*^*f/f*^ and *hTNFTg Cyld*^*M-Δ9/Δ9*^ mice at 6 weeks of age. **d** Weight differences among *Cyld*^*f/f*^, *hTNFTg Cyld*^*f/f*^ and *hTNFTg Cyld*^*M-Δ9/Δ9*^ mice at age of 4 and 8 weeks. **e** Histological examination of joints to evaluate inflammation (H/E panel), cartilage degradation (Toluidine blue-T/B panel) and bone erosions (Tartrate-resistant acid phosphatase-TRAP panel) in serial sections of 8-week old mice (*n* = 5–7 mice/group); Scale bar: 500 μm. **f** mCT-based imaging and histomorhometric indexes for *hTNFTg Cyld*^*f/f*^ and *hTNFTg Cyld*^*M-Δ9/Δ9*^ mice at age of 4 and 8 weeks (*n* = 9–13, week 4; *n* = 4–8, week 8). (BV/TV (%) bone volume fraction, Tb.Th. trabecular thickness, Tb.N trabecular number, Tb.Sp trabecular separation). Data are presented as the mean ± SEM. *p < 0.05, **p < 0.01, ***p < 0.001 and ****p < 0.0001 by two-tailed Student’s *t*-test (**e**, **f**).

To study the role of Cyld in SFs we generated mice carrying a mesenchymal-specific homozygous deletion of *Cyld* exon 9, by crossing mice containing floxed exon 9 of *Cyld* [30] with *Col6a1-Cre* transgenic mice [3]. Exon 9 deletion results in the expression of a truncated form of *Cyld* which lacks DUB activity but retains domains interacting with TRAF2 or NEMO [30]. The *Col6a1-Cre Cyld* ^*f/f*^ mice (from herein referred as *Cyld*^*M-Δ9/Δ9*^) were viable and fertile. The mice appeared normal in development with no apparent growth defects even at 12 months of age (data not shown). To evaluate the mesenchymal-specific contribution of Cyld in regulating the TNF-modeled arthritis, we generated mice of the *hTNFTg Cyld*^*M-Δ9/Δ9*^ genotype. The mice were viable and born in normal mendelian frequency. However, their growth was evidently retarded as early as 4 weeks of age, compared to non-Cre littermate controls (Fig. 1a). The gross observation of the mice at the age of 6 weeks revealed excessive swelling and redness of ankle joints (Fig. 1b) and deformity of wrinkle joints (Fig. 1c).

To further evaluate the effects of mesenchymal-specific Cyld inhibition on arthritic features, histological sections of ankle joints from all groups of mice were analyzed in early (week 4) and established *hTNFTg* disease (8 weeks). Four-week-old control *hTNFTg Cyld*^*f/f*^ mice showed moderate inflammatory synovitis and mild cartilage degradation as previously reported [31]. In sharp contrast, the *hTNFTg Cyld*^*M-Δ9/Δ9*^ mice developed severe inflammation of the synovium which was accompanied by marked proteoglycan loss of articular cartilage (Fig. 1e). In later stages (8 weeks), the *hTNFTg Cyld*^*f/f*^ mice developed full-blown manifestations of synovitis, cartilage destruction and bone erosion (Fig. 1e). At the same age, the histological findings in the ankle joints of *hTNFTg Cyld*^*M-Δ9/Δ9*^ mice suggested an extremely severe arthritic phenotype as this exhibited by the complete loss of joint architecture (Fig. 1e). The 3D histomorphometric analysis of calcaneus bone fully supported the histopathological findings, with all bone parameters being heavily affected (Fig. 1f).

**Fig. 2:**
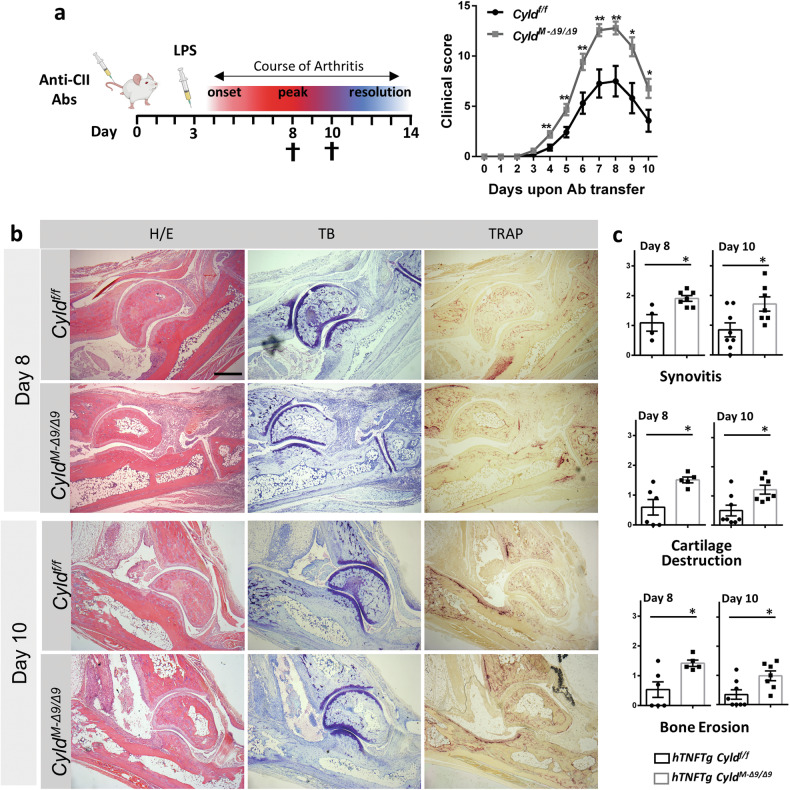
Mesenchymal Cyld-DUB deficiency exacerbates collagen-antibody-induced arthritis (CAIA). **a** CAIA was induced in mice by injection of an anti-collagen antibody cocktail on day 0, followed by a single injection of 75 µg of LPS on day 3 (left panel). Semiquantitative arthritis scores of *Cyld*^*f/f*^ and *Cyld*^*M-Δ9/Δ9*^ mice upon CAIA induction (right panel) (*n* = 14, two experiments). **b** Representative photos of ankle joint histology from mice at day 8 and day 10 upon CAIA induction. **c** Semiquantitative evaluation of inflammation (see **b**, H/E panel), cartilage degradation (see **b**, T/B panel) and bone erosions (see **b**, TRAP panel) in serial sections of mice (n = 7–8 mice/group); Scale bar: 500 μm. Data are presented as the mean ± SEM. *p < 0.05 and **p < 0.01.

To further confirm the role of mesenchymal Cyld-DUB deficiency in the development of arthritic phenotypes, we subjected *Cyld*^*M-Δ9/Δ9*^ and littermate *Cyld*^*f/f*^ control mice to CAIA [2] and we followed up the disease for 10 days (Fig. 2a). *Cyld*^*M-Δ9/Δ9*^ mice exhibited worsened arthritic manifestations both macroscopically and histologically at the peak of disease (day 8) and the remission phase (day 10), compared to Cyld-sufficient littermate controls (Fig. 2b). Collectively, these results clearly show that mesenchymal Cyld-DUB deficiency deteriorates arthritic manifestations in both innate- and autoimmune-based arthritis.

### Cyld-DUB deficiency predisposes SFs to generate an amplified inflammatory signature

**Fig. 3:**
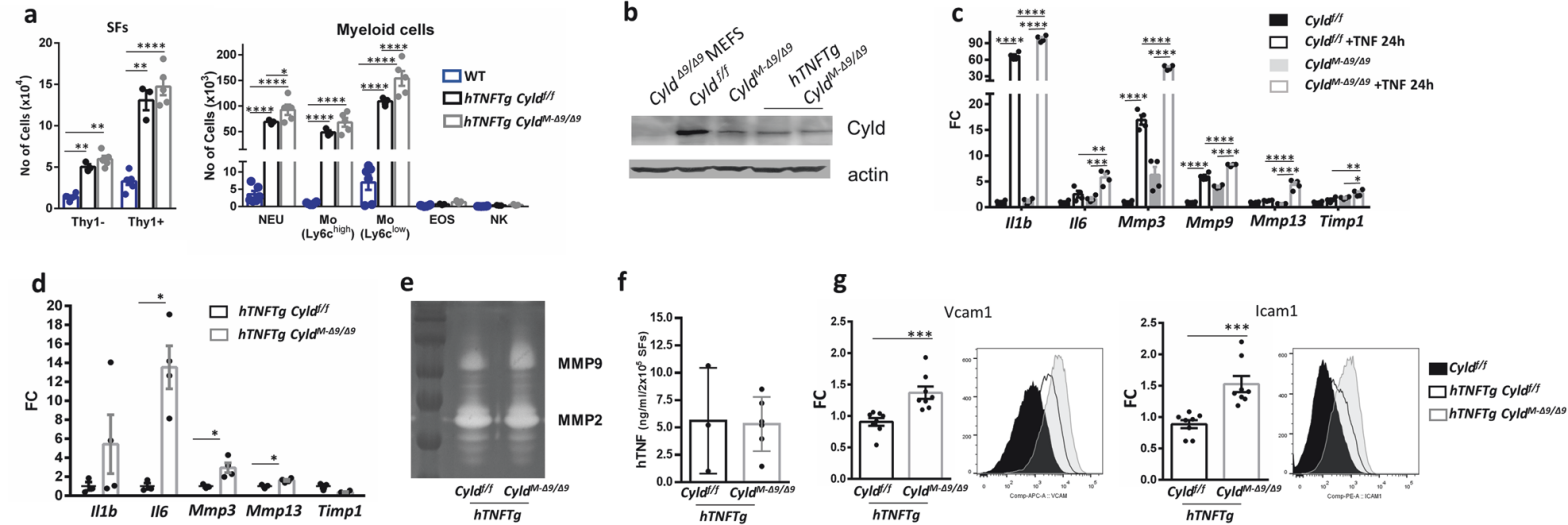
Cyld-DUB-deficient SFs exhibit enhanced expression of arthritic mediators. **a** Flow cytometric analysis of SF subtypes (Τhy1-, Lining SFs; Thy1+, Sublining SFs) and myeloid infiltrates in the ankle joints of *hTNFTg Cyld*^*f/f*^ and *hTNFTg Cyld*^*M-Δ9/Δ9*^ 4-week-old mice compared to WT controls. **b** Cyld expression in SF cultures derived from indicated genotypes. Extracts from Cyld-deficient MEFs served as control. **c** Relative gene expression of arthritic mediators *Il1b, Il6*, *Mmp3*, *Mmp9, Mmp13*, *and Timp1* (normalized to b2m levels) in SFs of Cyld-proficient and Cyld DUB-deficient SFs upon TNF (20 ng/ml, 24 h) (*n* = 4). **d** Relative gene expression of arthritic mediators *Il1b, Il6, Mmp3, Mmp13 and Timp1* (normalized to b2m levels) in SFs of *hTNFTg* Cyld-proficient and Cyld DUB-deficient SFs. **e** Representative gelatin-based zymography utilizing supernatants of SF cultures derived from hTNFTg Cyld-proficient and Cyld DUB-deficient SFs. **f** hTNF protein levels in cultures of *hTNFTg* Cyld-proficient and Cyld DUB-deficient SFs (*n* = 3–6). **g** Flow cytometric detection of Vcam-1 and Icam-1 levels in cultured *hTNFTg Cyld*^*f/f*^ (black), *hTNFTg Cyld*^*M-Δ9/Δ9*^ (gray) (upper panel) and representative FACS plots (*n* = 8, 2 experiments). Data are presented as the mean ± SEM (or mean ± SD for **f**) *p < 0.05, **p < 0.01, ***p < 0.001 and ****p < 0.0001 by two-way ANOVA (Bonferroni correction) (**a**) one-way ANOVA (Bonferroni correction) (**c**) or unpaired t test with Welch’s correction (**d**, **g**).

To better understand the effects of Cyld-DUB deficiency in the deterioration of arthritis, we analyzed major cellular composites characterizing arthritic joints during the early phase of *hTNFTg* arthritis (4 weeks of age). The relative abundance of lining (Thy1-) and sublining (Thy1+) synovial fibroblast types did not significantly differ between the *hTNFTg Cyld*^*M-Δ9/Δ9*^ and *hTNFTg Cyld*^*f/f*^ genotypes, however, both genotypes showed statistical differences compared to normal SFs (Fig. 3a-left panel, Supplementary Fig. 1). The analysis of the immune content at the same age highlighted higher numbers of neutrophils and Ly-6C^low^ monocytes in the *hTNFTg Cyld*^*M-Δ9/Δ9*^ joints compared to *hTNFTg Cyld*^*f/f*^ controls (Fig. 3a-right panel). Interestingly, neutrophils and Ly-6C^low^ monocytes are crucial for the initiation of arthritic phenotype and their infiltrating level reflects the severity of disease [32, 33]. Therefore, the analysis of cellular content of diseased joints suggests that the enhanced immune infiltrations in *hTNFTg Cyld*^*M-Δ9/Δ9*^ joints, rather than overrepresentation of SFs, characterize the accelerated disease onset.

To determine how Cyld-DUB-deficient SFs affect the recruitment and the tissue destruction in the arthritic joints, we examined SF responses ex vivo. Reduction of Cyld levels in cultured SFs isolated from ankle joints of both genotypes (Cre and non-Cre littermates) (Fig. 3b) is consistent with the recombination efficacy reported for the *Col6a1-Cre* transgenic mice [3]. We then analyzed gene expression of known regulators of arthritic disease. TNF-stimulated Cyld-DUB-deficient SFs were highly responsive to TNF signals compared to the Cyld-sufficient SFs, expressing higher levels of cytokines and proteases (Fig. 3c). Interestingly, the levels of *Mmp9* mRNA in Cyld-DUB-deficient SFs were higher even in naïve conditions. Similarly, *hTNFTg* Cyld-DUB-deficient cells showed a particularly high expression profile of *IL-6* as well as disbalanced expression ratio of the proteases *Mmp3* and *Mmp13* (Fig. 3d). Moreover, supernatants of the *hTNFTg* Cyld-deficient SF cultures showed higher MMP9 activity when analyzed by zymograms (Fig. 3e). Remarkably, the expression levels of *hTNF* transgene remained unaltered (Fig. 3f). Expression of adhesion molecules VCAM-1 and ICAM-1 were elevated in *hTNFTg* Cyld-DUB-deficient SFs compared to *hTNFTg* Cyld-sufficient cells (Fig. 3g). These results suggest that Cyld-DUB regulates gene expression upon inflammatory stimuli in SFs and correlate well with the exacerbated arthritic phenotype of *hTNFTg Cyld*^*M-Δ9/Δ9*^ mice.

### Cyld-DUB deficiency causes aberrant TNF-mediated signaling responses in SFs

**Fig. 4:**
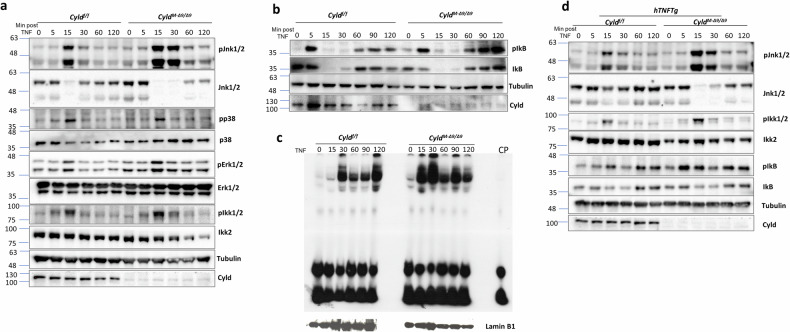
Cyld-DUB-deficient SFs exhibit altered TNF-mediated signaling outputs. **a** Western blot analysis for the detection of MAPK/SAPK and IKK activation in *Cyld*^*f/f*^ and *Cyld*^*M-Δ9/Δ9*^ cultured SFs upon TNF stimulation (10 ng/ml) for the indicated time points. (*n* = 5). **b** Immunoblot for the detection of IkB phosphorylation and degradation pattern upon TNF stimulation (10 ng/ml) (*n* = 3). **c** Band shift assay for the detection of NF-κB binding activity of nuclear extracts derived from *Cyld*^*f/f*^ and *Cyld*^*M-Δ9/Δ9*^ SF cultures, upon TNF stimulation for the indicated time points (*n* = 3). (CP cold probe). The same amount of lysates was subjected to Western blot analysis of LaminB1 expression. **d** Western blot analysis for the detection of JNK, IKKs, and NF-κB activation in *hTNFTg Cyld*^*f/f*^ and *hTNFTg Cyld*^*M-Δ9/Δ9*^ cultured SFs upon TNF stimulation (10 ng/ml) for the indicated time points (*n* = 5).

We analyzed signaling events that underline the arthritic gene expression in Cyld-DUB-deficient SFs. As previously reported for Cyld-DUB-deficient MEFs [30], the Cyld DUB-null SFs exhibited apparent aberration in NF-κB and JNK responses upon TNF stimulation, characterized by enhanced and prolonged phosphorylation of Jnk1/2 kinases, as well as IKK2 kinase followed by altered IkB phosphorylation pattern (Fig. 4a, b, Supplementary Fig. 2a, b, d). Interestingly, ERK and p38 activation was less affected (Fig. 4a, Supplementary Fig. 2a). Consistently, the nuclear extracts from Cyld deficient SFs exhibited increased DNA binding activity to NF-κB binding sites compared to Cyld-proficient SFs (Fig. 4c). We also confirmed the aberrant activation of JNK and NF-κB in arthritic Cyld DUB-null SFs (Fig. 4d, Supplementary Fig. 2c). Collectively, the above results demonstrate that, upon TNF stimulation, Cyld DUB inactivation in SFs causes enhanced NF-κB and JNK responses, suggesting that Cyld-DUB deficiency renders SFs hypersensitive to TNF-mediated signals.

### Mesenchymal Cyld targets TAK1/IKK2 axis to regulate proinflammatory SF responses in TNF-mediated arthritis

**Fig. 5:**
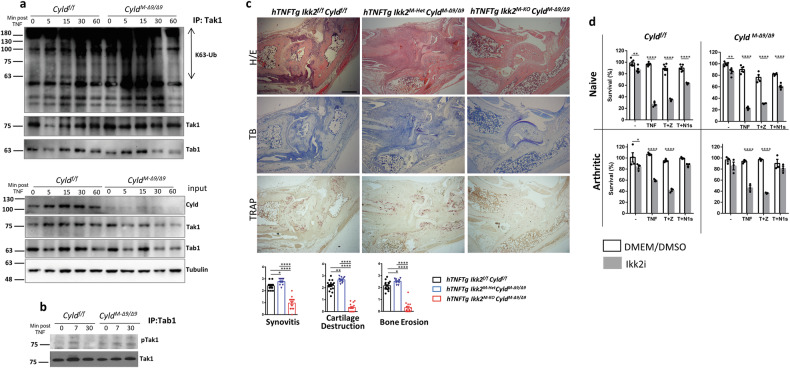
The TAK1/IKK2 axis regulates TNF arthritogenicity in TNF-mediated arthritis. **a** Detection of Tak1 ubiquitination by immunoassay of *Cyld*^*f/f*^ and *Cyld*^*M-*^^*Δ9/Δ9*^ SFs stimulated with TNF (0–60 min), followed by immunoprecipitation of lysates with anti-Tak1 and immunoblot analysis with anti-K63-Ub, and then re-probing with anti-Tak1 and anti-Tab1. (Lower panel) Immunoblot analysis of corresponding whole cell lysates (input) with anti-Cyld, anti-Tak1 and anti-Tab1 (*n* = 3). **b** Detection of Tak1 phosphorylation by immunoassay of *Cyld*^*f/f*^ and ^*CyldM-Δ9/Δ9*^ SFs stimulated with TNF (0–30 min), followed by immunoprecipitation of lysates with anti-Tab1 and immunoblot analysis with anti-pTak1. **c** Histological evaluation *of 10-week-old hTNFTg Ikk2*^*f/f*^ *Cyld*^*f/f*,^ *hTNFTg Ikk2*^*M-Het*^*Cyld*^*Μ-Δ9/Δ9*^ and *hTNFTg Ikk2*^*M-KO*^*Cyld*^*Μ-Δ9/Δ9*^ mice; Scale bar: 500 μm. **d** Survival rates of *Cyld*^*f/f*^ and *Cyld*^*M-Δ9/Δ9*^ SFs treated either with 50 ng/ml TNF or combinations of TNF with zVAD or Nec1 as indicated (T + Z: TNF 50 ng/ml + zVAD, T + N1s: TNF 50 ng/ml+ Nec1s) in the absence (Black bars) or presence (Gray bars) of ΙΚΚ2 inhibitor ML120b, s (*n* = 3–5). Data are presented as the mean ± SEM *p < 0.05, **p < 0.01, and ****p < 0.0001 by one-way ANOVA (Bonferroni correction) (**c**) or two-tailed Student’s *t*-test (**d**).

To determine the molecular basis of aberrant TNF-mediated NF-κB and JNK responses due to Cyld-DUB deficiency, we sought to examine the activity of Tak1. Tak1 is implicated in both TNF-induced NF-κB and JNK pathways, and its kinase activity is regulated by Cyld-mediated deubiquitination of K63-Ub chains [20]. We reasoned that the prolonged ubiquitination of Tak1 due to Cyld-DUB deficiency in SFs, leads to the sustained phosphorylation of IKK2 and Jnk (Fig. 4a, d). The pattern of K63 ubiquitination of Tak1 following TNF stimulation differed between Cyld-sufficient and Cyld-targeted SFs. K63 ubiquitination of Tak1 was evident even in unstimulated conditions in Cyld-targeted SFs and was significantly higher in later time points compared to Cyld-sufficient SFs (Fig. 5a, Supplementary Fig. 2e). Consistently, Cyld-DUB-deficient SFs exhibited sustained Tak1 phosphorylation compared to control SFs (Fig. 5b).

Interestingly, neither Jnk1 nor Jnk2 deficiency was sufficient to modify the *hTNFTg* disease ([34] and Suppl Fig. 3 respectively). However, mice with mesenchymal -specific Cyld/IKK2 deficiency in the *hTNFTg* background (*hTNFTg Ikk2* ^M-KO^*Cyld*^M-Δ9/Δ9^) exhibited modified and attenuated arthritic phenotype compared to their littermate Ikk2/Cyld-sufficient controls (Fig. 5c), resembling the phenotype of *hTNFTg Ikk2*^M-KO^ mice [2]. This finding indicates that dominant IKK2-mediated signaling is responsible for the exaggerated arthritic response in *hTNFTg Cyld*^M-Δ9/Δ9^ mice.

Taken together, these results argue against a sufficient role of JNK1/2 kinase activation for the development of hTNF-mediated disease and indicate that the prolonged activation of TAK1 due to Cyld-DUB deficiency in SFs promotes a prolonged activation of IKK2 and NF-κB (Fig. 4a, d), leading to exacerbated arthritogenic responses in vivo.

### Cyld is insufficient to control the TNF-induced death of SFs

Previous evidence demonstrated that Cyld is implicated in the regulation of TNF-mediated programmed necrosis through the deubiquitination of Ripk1, abrogating the formation of the Ripk1/Ripk3 complex which activates the necroptosis executor kinase Mlkl [27]. In the context of *hTNFTg* arthritis, complete absence of Ripk3 kinase does not affect development of arthritis, indicating that Ripk3-mediated signals are not directly implicated in the pathology. However, Ripk3 deficiency rescues residual synovial inflammation of *hTNFTg Ikk2*^*M-KO*^ mice by restricting inflammatory SF death caused by the absence of SF-IKK2 kinase [2]. Herein, we additionally illustrate that the residual inflammation of *hTNFTg Ikk2*^*M-KO*^ mice is fully restored by the deficiency of Mlkl (Supplementary Fig. 4). This finding strongly suggests that the remaining inflammatory activity in *hTNFTg Ikk2*^*M-KO*^ mice is caused by necroptosis of IKK2-null SFs. In sharp contrast, SF-Cyld DUB deficiency was not sufficient to abrogate residual disease of *hTNFTg Ikk2*^*M-KO*^ mice (Fig. 5c) also compare the age-matched *hTNFTg Ikk2*^*M-KO*^ *Mlkl*^*-/-*^ phenotype (Supplementary Fig. 4) to *hTNFTg Ikk2*^*M-KO*^ *Cyld*^*M-Δ9/Δ9*^ and littermate control mice). Consistent with the in vivo phenotype of *hTNFTg* mesenchymal-specific *Cyld/Ikk2* mutants, TNF-induced cytotoxicity in Cyld-proficient and deficient SFs exhibited similar survival outcomes either in the presence or absence of IKK2 activity and caspases. The addition of Nec1s (Ripk1 inhibition), however, was sufficient to rescue lethality by TNF and concomitant IKK2 inhibition in SFs of all examined genotypes [2] (Fig. 5d). This finding indicates that Cyld is redundant for the regulation of the TNF-induced, Ripk1-mediated death of IKK2-null SFs and, consequently, the necroptosis-mediated residual disease in the *hTNFTg* mesenchymal -specific Ikk2 mutants.

Collectively, our results show that SF-Cyld controls TAK1 kinase to prevent IKK2 and NF-κB hyperactivation but not Ripk1-mediated death, leading to deterioration of arthritic phenotype in TNF-dependent arthritis.

## Discussion

Compelling evidence suggests important contributions of Cyld-mediated deubiquitination in signaling pathways involved in development, homeostasis and several pathologies [8], but less is known about the role of CYLD in inflammatory arthritis. While CYLD is significantly downregulated in human-cultured RASFs compared to Osteoarthritic SFs [29], mechanistic insights into the role of the SF-CYLD are still missing. It is well-acknowledged that the persistent inflammation in RA entails a positive feedback whereby activated SFs recruit and retain immune cells to the joint, causing both cell types to shape and respond to the microenvironment in a self-sustaining manner [35]. Here we provide evidence that underlines CYLD in SFs as a robust modulator of inflammatory arthritis that functions to coordinately limit excessive activation of inflammatory and destructive properties of SFs. We show that mesenchymal Cyld contributes to prevent excessive JNK and NF-κB activation in TNF-stimulated naive and arthritic SFs. Accordingly, high transcriptional activity of proteases and cytokines involved in the arthritic process accompanied the deteriorated phenotype of mesenchymal Cyld DUB-deficient mice. These effects were observed both in genetic and induced models of arthritis. In sharp contrast, the elimination of Cyld DUB activity in the mesenchymal tissues (Col6a1-Cre+) is not detrimental for homeostasis.

The molecular analysis of Cyld DUB activity in limiting activation of NF-κB and JNK in TNF-treated SFs highlights a common denominator of the two pathways, the Tak1 kinase. Cyld-DUB-deficient SFs exhibit Tak1 hyperactivation, leading to sustained IKK2 and IkB phosphorylation and, eventually, deregulated activation of NF-κB. Indeed, *hTNFTg* mice with concomitant mesenchymal Cyld/Ikk2 mutation display amelioration of arthritogenic responses, phenocopying the *hTNFTg Ikk2*^*M-KO*^ mice [2]. Even though JNK/MAPK pathway is also deregulated in Cyld-deficient SFs, our results and previous studies argue against a dominant pathogenic role of either JNK2 or JNK1 in TNF-mediated arthritis [34], without excluding, however, their synergistic role in disease. Moreover, other mechanisms, including accumulation of reactive oxygen species (ROS), may also contribute to increased JNK phosphorylation [36, 37]. Consistent with our findings on TAK1 hyperactivation, previous pharmacological studies have identified TAK1 as a central signal transducer that mediates the pathogenic activation of SFs ex vivo and the arthritic outcome in vivo [38, 39]. The embryonic lethality of mesenchymal double genetic Cyld/Tak1 mutant mice and the absence of inducible SF-specific Cre lines have not allowed us to further analyze the mesenchymal Cyld/TAK1 crosstalk in arthritis, as previously exhibited for hepatocytes [26, 28].

In the context of the role of Cyld-DUB in cell death responses of SFs, the concomitant mesenchymal Cyld/Ikk2 deficiency under *hTNFTg* conditions additionally suggested the redundant function of Cyld in regulating the de novo RIPK3/MLKL-dependent necroptosis detected in IΚΚ2-targeted arthritic SFs in vivo and ex vivo [2]. This finding contrasts current concepts on the dominance of Cyld-DUB activity in regulating necroptosis. Limited evidence supports our contradictory findings, as the RIPK3-mediated death observed in the colon of intestinal epithelial NEMO deficiency was not rescued by Cyld DUB deficiency [40], while the repression of Cyld in neuronal cells promoted cell death but did not alter NF-κB activity upon brain injury [41]. Interestingly, several studies corroborate the context-specific effects of complete Cyld protein deficiency in different cell types, such as gingival fibroblasts in periodontal diseases, where TNF and LPS exerted differential NF-κB outcomes [42] or in Cyld-deficient macrophages and T-cells which differentially respond to innate (TNF, LPS) or adaptive (anti-CD3) stimuli respectively [43, 44]. However, we cannot fully dismiss that incomplete Cyld targeting, dominant-negative functions of truncated Cyld [28], other spatiotemporal signaling events [45] or context-specific signaling counterparts of Cyld (Itch1, usp18, USP4, spata2) may account for the insufficiency of Cyld to prohibit necroptosis in our experiments with the IKK2-prohibited SFs.

Likewise, it is also tempting to consider that K63 deubiquinating activity of A20 cannot reconstitute for the Cyld insufficiency in SFs, indicating that the crucial target of Cyld in SFs could be other than the common set of targets such as TRAF2, TRAF6, RIPK1, and NEMO [8]. It was previously suggested that A20 and Cyld differentially act to regulate TNF pathway; A20 expression is induced by inflammatory stimuli and acts to terminate responses [46, 47] while Cyld is constitutively active in order to prohibit uncontrolled NF-κB activation [48]. Alternatively, a prominent role of Cyld-mediated M1 deubiquitination in TNF signaling in SFs remains to be explored [49, 50].

Collectively, by exhibiting the major contribution of Cyld deubiquitinase activity in the TAK1/IKK2 axis leading to pathogenic NF-κB activation, we identify Cyld as a negative regulator of the major inflammatory signaling acting downstream of TNFR1 in SFs. Our study, therefore, contributes to the signaling map underlying SF responses in TNF-mediated arthritis that would inform potential therapeutic - strategies targeting SFs.

## Material and methods

### Animals and induction of arthritis

Human TNF transgenic (*hTNFTg*) [31], *TgCol6a1-Cre* [3], *Cyld* ^*fxl9/flx9*^ (referred in the text and figures as *Cyld*^*f/f*^), Milk^−/−^ [51] and *Ikbkb*^*f/f*^ [52] (referred in the text and figures as *Ikk2*^*f/f*^) mice have been previously described. *TgCol6a1-Cre* *Cyld*^*flx9/flx9*^ mice referred as *Cyld*^*M-Δ9/Δ9*^. Collagen-Antibody-Induced Arthritis (CAIA) was induced according to the manufacturer’s instructions (MD Biosciences). Mice were maintained on a C57BL/6J genetic background.

### Clinical assessment of arthritis

Arthritis in *hTNFTg* animals was evaluated in ankle joints in a blinded manner using a semiquantitative arthritis score ranging from 0 to 4; 0: no arthritis (normal appearance and grip strength); 1: mild arthritis (joint swelling); 2: moderate joint swelling and digit deformation, reduced grip strength; 3: severe joint swelling and digit deformation, impaired movement, no grip strength; 4: severe joint swelling and digit deformation, impaired movement, no grip strength and obvious weight loss-cachexia. At indicated weeks of age, mice were sacrificed and the hind ankle joints were removed for histology analysis. CAIA clinical evaluation was performed as previously described [53].

### Histologic analysis

Formalin-fixed, EDTA-decalcified, paraffin-embedded mouse tissue specimens were sectioned and stained with hematoxylin and eosin (H/E), Toluidine Blue (TB) or TRAP kit [Sigma-Aldrich]. H&E-stained joint sections were semi-quantitatively blindly evaluated for the following parameters: synovial inflammation/ hyperplasia (scale of 0–4), cartilage erosion (scale of 0–4), and bone loss (scale of 0–4) [54].

### Microcomputed tomography

Microcomputed tomography (mCT) of excised joints was carried out by a SkyScan 1172 CT scanner (Bruker, Aartselaar, Belgium) following the general guidelines used for assessment of bone microarchitecture in rodents using mCT [55]. Briefly, scanning was conducted at 50 kV, 100 mA using a 0.5-mm aluminum filter, at a resolution of 6 μm/pixel. Reconstruction of sections was achieved using the NRECON software (Bruker) with beam hardening correction set to 40%. The analysis was performed on a spherical volume of interest (VOI) (diameter 0.53 mm) within 62 slices of the trabecular region of calcaneus. Morphometric quantification of trabecular bone indices such as trabecular bone volume fraction (BV/TV; %), trabecular thickness (Tb. Th; mm), trabecular number (Tb. N; 1/mm) and trabecular separation (Tb. Sp; mm) were performed using the CT analyzer program (Bruker).

### Flow cytometric analysis of joint tissue

The ankle joint tissues were disaggregated by incubation for 40 min at 37 °C in an enzymatic digestion medium consisting of DMEM, 10% heat-inactivated FBS, collagenase type IV (300 units) from *Clostridium histolyticum* [Sigma] and 0.03 mg ml^−1^ DNase [Sigma]. The cells were then blocked in 1% BSA in PBS and Fc blocker (anti-CD16/32) for 10 min at 4 °C. For SF subtype determination, the following fluorophore-conjugated antibodies were employed: anti-Pdpn PE/Cyanine7; anti-Thy1 Alexa Fluor 647; anti-CD31 APC/Fire™ 750; anti-CD45 APC/Cyanine7; anti-Ter119 APC/Fire™ 750 (Supplementary table 1). For myeloid infiltrates, we used the following antibodies: anti- Ly-6C FITC; anti-NK-1.1 PE; anti- CD11c PE/Dazzle™ 594; anti- Ly-6G PE/Cyanine7; anti-CD11b APC; anti- CD45 APC/Cyanine7. The analysis was performed with BD FACSCanto II and the BD FACSDiva software and dead cells were excluded by Propidium Iodide staining. Analysis of the results were performed employing FlowJo software (v.10)

### Cell culture

Primary mouse SFs were isolated from mice with indicated genotypes and cultured for four passages as previously described [56]. Upon evaluation of purity (less of 5% CD45+ cells in the culture), SFs were used in the assays. Flow cytometric analysis was performed in cells stained with the following antibodies CD90.2, VCAM-1, CD45, ICAM-1, and CD45 using FlowJo analysis software.

In experiments with IKK2 inhibitor, pretreatment of cells with ML120B (Tocris, 10uM) [57] was performed for 3 h before the addition of other reagents. zVAD [Bachem] and Nec1s [BioVision] were used in concentrations 20 and 30 mM, respectively. TNF cytotoxicity assays were performed in 96-well plates, terminated 16–18 h post TNF addition, and evaluated upon crystal violet staining, solubilization in 33% acetic acid solution and determination of optical density at 570 nm as a test filter and 630 nm as a reference filter.

### Quantitative PCR

The RNA extraction was performed employing TRIZOL (Thermo). The extracted RNA was further treated with DNAse to eliminate DNA residues (Quigen, RNeasy MinElute cleanup kit). Upon RT reaction using the MMLV Reverse Transcriptase (Promega), according to the manufacturer’s instruction. The cDNA was further subjected to qPCR (ThermoFisher Scientific, Platinum™ SYBR™ Green qPCR SuperMix) for the detection of *Il1b*, *Il6*, *Mmp3*, *Mmp9, Mmp13* and *Timp1* (oligos provided in Supplementary Table 2) in a CFX96 Touch Real-Time PCR Detection System (Biorad). Quantification was performed with the ddCt method.

### Immunoblotting and Immunoprecipitation

For immunoblotting, samples were collected in RIPA buffer, containing 1% Triton X-100, 0.1% SDS, 150 mM NaCl, 10 mM Tris–HCl, pH 7.4, 1 mM EDTA, protease inhibitors [Roche], and phosphatase inhibitors [Sigma-Aldrich], separated by SDS/PAGE (10–12.5%), transferred to nitrocellulose membranes [Millipore], and probed with antibodies (Supplementary Table 1). Chemiluminescent signals were detected by the Bio-Rad Gel Doc EZ imaging system or by conventional methods.

For immunoprecipitation experiments, SFs were stimulated with murine TNF (20 ng/ml) (VIB Protein Service Facility) as indicated. Cells were lysed in Triton X-100 buffer (50 mM Tris–HCl pH 7.5, 1% Triton X-100, 1 mM EDTA pH 8, 1 mM EGTA pH 8, 250 mM Sucrose, protease inhibitors [Roche] and phosphatase inhibitors [Sigma-Aldrich]), and the extracts were incubated either with 2ug anti-Tak1 [Cell Signaling] or 2 μg anti-Tab1 antibody [58] (kindly provided by Philip Cohen/University of Dundee) for 4 h. Protein A/G beads [Santa Cruz Biotechnology] were added for the 3 h of incubation. The beads were then washed three times and the samples were eluted from the beads using Laemni buffer, separated by SDS/PAGE (10%), transferred to nitrocellulose membranes and subjected to immunoblot, as described, utilizing appropriate antibodies (Supplementary table 1)

### EMSA

To generate dsDNA with NF-κB binding site, we mixed appropriate oligos with complementary sequences (200 ng each) (sense5′-ATCAGGGACTTTCCGCTGGGGACTTT-3′ and antisense5′CGGAAAGTCCCCAGCGGAAAGTCCCT-3′), heated to 100 °C, and allowed to return slowly to room temperature. Upon ^32^P-labeleling, the ds DNA fragments with the NF-κB binding site were incubated with nuclear extract (5 μg per reaction) in 25 mM HEPES-KOH at pH 7.9, 250 mM KCl, 25 mM MgCl_2_, 2.5 mM DTT, 50% glycerol, at room temperature for 20 min. After addition of 20% Ficoll, samples were subjected to electrophoresis on a 6% polyacrylamide gels, run with 50 mM Tris-borate buffer pH 8.3, 1 mM EDTA at 4^o^C. Dried gels were visualized by phospho-imaging using Personal Molecular Imager FX system (Bio-Rad Laboratories) or films.

### Statistical analysis

Data are presented as mean ± SEM. Analyses were performed by employing SigmaPlot and GraphPad software. *p* values < 0.05 were considered significant.

### Supplementary information

Supplementary Figures

Supplementary Tables

Uncropped Immunoblots

### Source data

Source Data

## Data Availability

The data that support the findings of this study are available from the corresponding author upon reasonable request.
