# Supplementary material for: Cyld restrains the hyperactivation of synovial fibroblasts in inflammatory arthritis by regulating the TAK1/IKK2 signaling axis
Source: Cell Death Dis. 2024 Aug 9;15(8):584. doi: 10.1038/s41419-024-06966-2 (PMC11316070; doi:10.1038/s41419-024-06966-2)
Supplement: Supplementary file 1 — Supplementary Figures [file 41419_2024_6966_MOESM1_ESM.pdf]

Supplementary Figure 1 (relative to Figure 3)

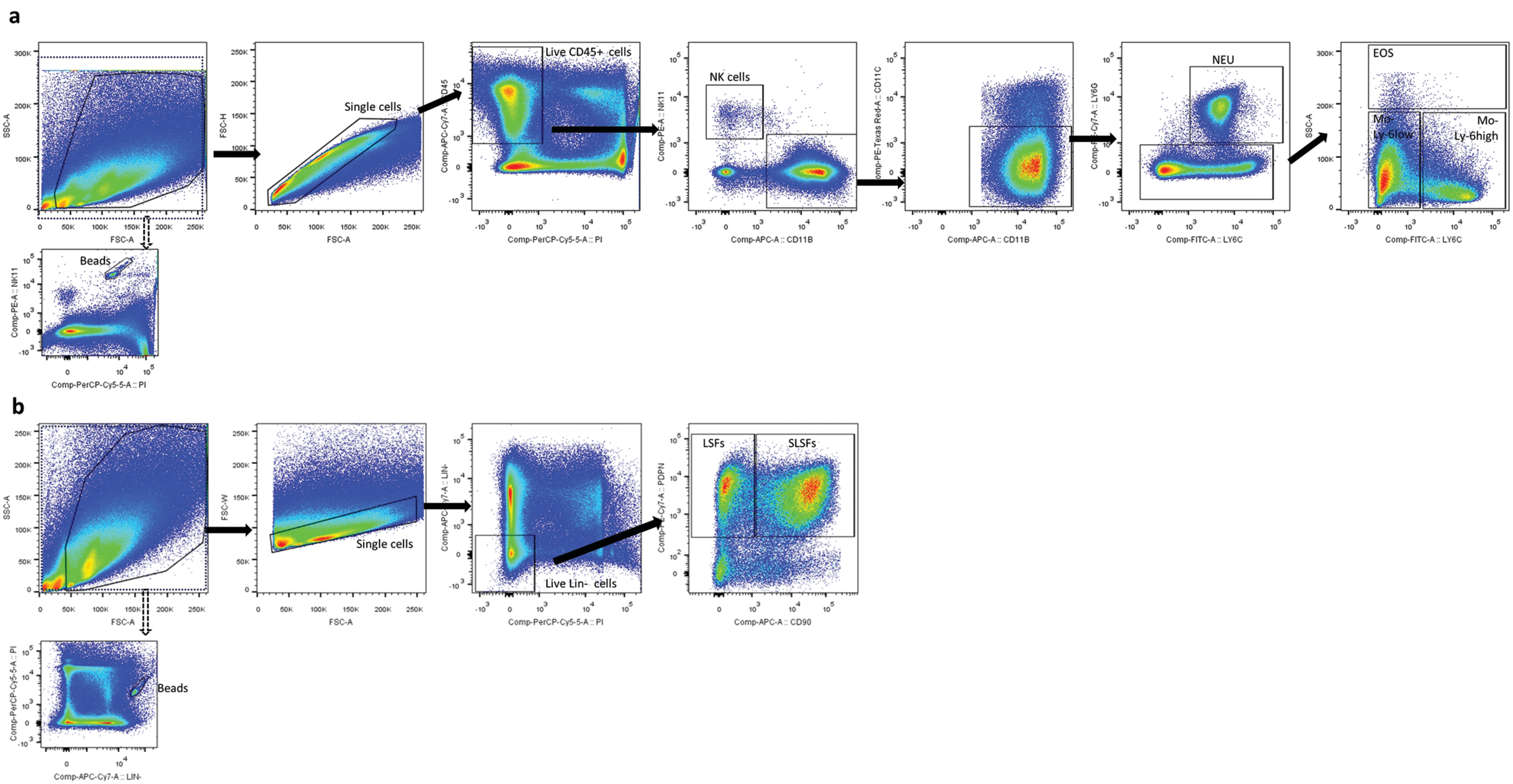

Suppl. Figure 1. Gating strategy as applied for immune and fibroblast cell populations described in Fig. 3A. (a) Non debris, singlets, live, CD45+ CD11b− NK1.1+ (NK cells), CD45+, CD11b+, NK1.1-, CD11c-, Ly-6G+ (Neutrophils, NEU), CD45+, CD11b+, NK1.1-, CD11c-, Ly-6C high and low monocytes (Mo) and CD45+, CD11b+, NK1.1-, CD11c-, SSC high cells (Eosinophils, EOS); (b) Non debris, singlets, live, lineage− (CD45-, CD31-, Ter119-), Pdpn+/Thy1.2- (CD90.2) fibroblasts (LSFs) and Pdpn+/Thy1.2+ (CD90.2) fibroblasts (SLSFs).

Supplementary Figure 2 (relative to Figure 4)

a

	Cyld$^{f/f}$						*Cyld*$^{M\text{-}\Delta9/\Delta9}$					
	0	5	15	30	60	120	0	5	15	30	60	120
pJNK2	1.00	1.87	10.65	4.35	1.87	1.24	2.88	4.71	17.72	19.77	7.04	3.62
pJNK1	1.00	1.11	3.48	1.73	0.91	0.81	1.40	1.69	2.81	5.16	2.23	1.59
pp38	1.00	1.62	6.48	4.65	1.86	1.55	1.60	2.23	4.01	1.09	0.77	0.86
pERK1	1.00	1.65	3.43	1.81	1.71	1.59	2.60	2.79	4.91	2.57	1.97	1.73
pERK2	1.00	1.06	2.14	0.96	0.77	0.75	1.09	1.13	1.89	1.45	1.11	1.10
pIkk1/2	1.00	2.98	6.97	2.37	1.76	1.45	1.00	3.61	11.45	6.30	3.73	3.17
Cyld	1.00	1.30	1.09	1.15	0.82	0.89	0.18	0.17	0.23	0.19	0.26	0.19

b

	Cyld$^{f/f}$							*Cyld*$^{M\text{-}\Delta9/\Delta9}$						
	0	5	15	30	60	90	120	0	5	15	30	60	90	120
pIkBa	1.00	12.14	0.98	0.67	3.58	4.53	5.18	1.69	11.00	1.40	1.53	10.57	23.03	30.51
IkBa	1.00	0.79	0.03	0.09	0.56	0.44	0.37	0.42	0.28	0.02	0.07	0.68	0.96	1.69
Cyld	1.00	1.91	1.02	1.48	0.83	0.75	0.97	0.07	0.26	0.18	0.21	0.19	0.17	0.18

c

	hTNFTg											
	Cyld$^{f/f}$						*Cyld*$^{M\text{-}\Delta9/\Delta9}$					
	0	5	15	30	60	120	0	5	15	30	60	120
pJNK2	1.00	1.51	6.43	3.48	2.15	2.42	1.77	2.89	19.77	13.29	2.80	4.26
pJNK1	1.00	0.84	2.50	1.56	1.20	1.20	1.02	1.88	7.43	5.01	1.68	3.98
pIkk1/2	1.00	1.55	5.21	2.28	1.55	1.55	1.66	3.51	9.81	4.85	4.19	3.61
pIkBa	1.00	1.30	2.23	1.18	2.45	2.56	2.18	6.26	4.71	3.12	2.84	4.66
IkBa	1.00	1.13	0.82	0.58	1.39	1.62	1.42	1.98	1.25	1.23	1.33	2.70
Cyld	1.00	1.13	1.11	0.89	1.15	0.95	0.18	0.19	0.20	0.19	0.08	0.10

d

IkB phosphorylation and degradation pattern
(comparison per genotype)

		Cyld$^{f/f}$							*Cyld*$^{M\text{-}\Delta9/\Delta9}$						
		0	5	15	30	60	90	120	0	5	15	30	60	90	120
Non-arthritic	pIkBa	1.00	12.14	0.98	0.67	3.58	4.53	5.18	1.00	6.51	0.83	0.90	6.25	13.62	18.05
	IkBa	1.00	0.79	0.03	0.09	0.56	0.44	0.37	1.00	0.67	0.05	0.16	1.60	2.28	4.00
hTNFTg	pIkBa	1.00	1.30	2.23	1.18	2.45	-	2.56	1.00	2.88	2.16	1.44	1.30	-	2.14
	IkBa	1.00	1.13	0.82	0.58	1.39	-	1.62	1.00	1.39	0.88	0.86	0.93	-	1.90

e

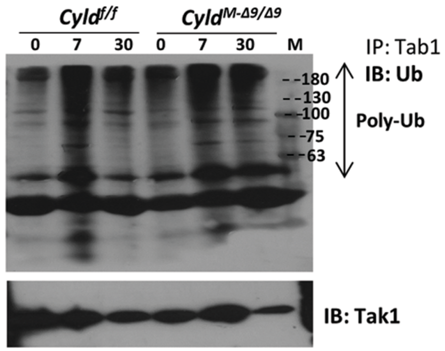

Suppl. Figure 2. (related to Figure 4 and 5) Quantitation of immunoblots. (a) Data generated from blots of Fig. 4a. Quantitation of pERK1/2 and pIKK were relative to total ERK1/1 and IKK2 respectively. Relative levels of pJNK1/2 were quantified to tubulin levels due to bleaching effect in all membranes tested. (b) Data generated from blots of Fig. 4b. Quantitation of pIkBa and IkBa were calculated to tubulin levels. (c) Data generated from blots of Fig. 4d. Quantitation of pERK1/2 and pIkk1/2 were relative to total ERK1/1 and IKK2 respectively. Relative levels of pJNK1/2 were quantified to tubulin levels. In all cases (a), (b) and (c) we set as reference quantity 1 the quantity of *Cyld*$^{f/f}$ 0min sample. (d) Relative levels of pIkBa and IkBa relative to time point 0min for each genotype. (e) Detection of Tak1 ubiquitination by immunoassay of *Cyld*$^{f/f}$ and *Cyld*$^{M\text{-}\Delta9/\Delta9}$ SFs stimulated with TNF (0-30min), followed by immunoprecipitation of lysates with anti-Tab1 and immunoblot analysis with anti-Ub, and then re-probing with anti-Tak1.

Supplementary Figure 3

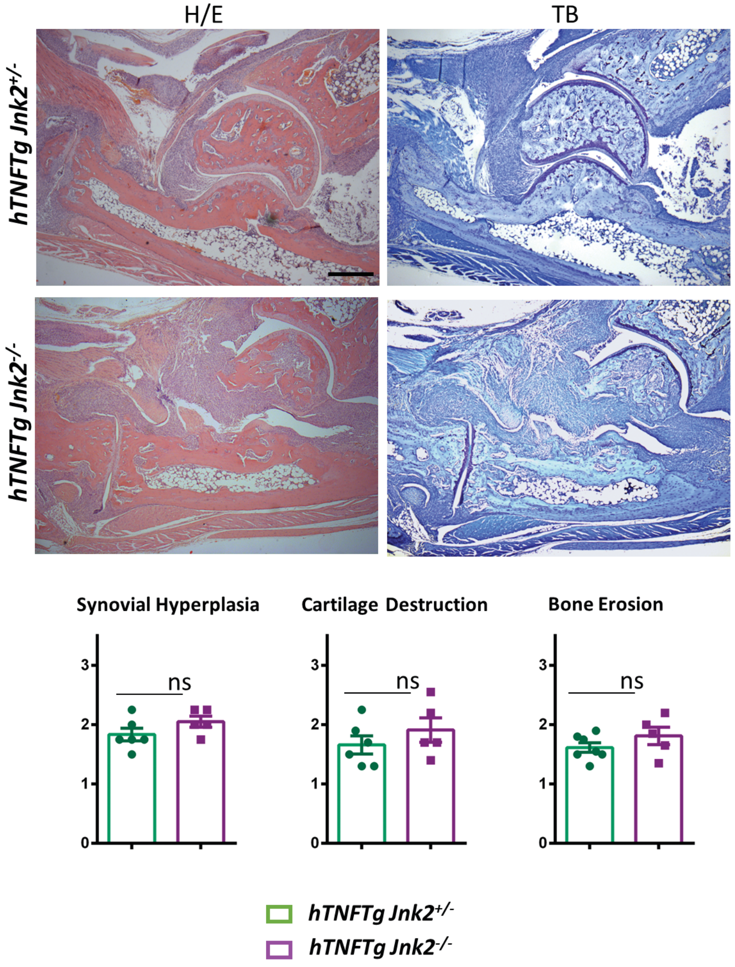

Suppl. Figure 3. Jnk2 does not critically regulate the arthritic phenotype of *hTNFTg* mouse. Histological evaluation of *hTNFTg Jnk2*$^{+/-}$ and *hTNFTg Jnk2*$^{-/-}$ mice with H/E and toluidine blue staining (n=5-6) at the age of 8 weeks; Scale bar: 500 µm. Data are presented as the mean ± SEM by two-tailed Student's t-test.

Supplementary Figure 4

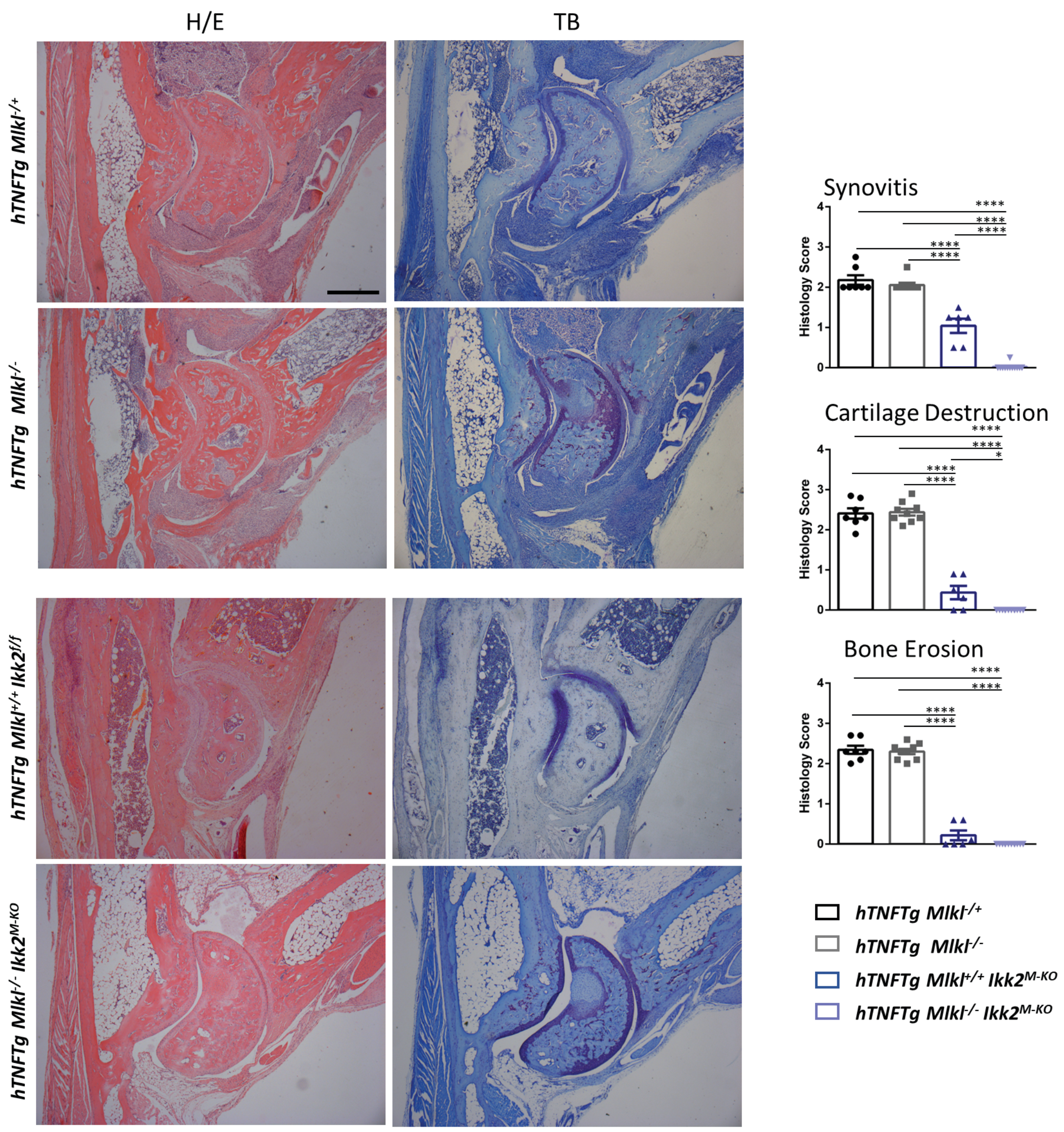

Suppl. Figure 4. Mlkl is necessary to prevent residual disease in *hTNFTg Ikk2*$^{M\text{-}KO}$ mice. Histological evaluation of *hTNFTg Mlkl*$^{+/-}$, *hTNFTg Mlkl*$^{-/-}$, *hTNFTg Mlkl*$^{+/+}$ *Ikk2*$^{M\text{-}KO}$ and *hTNFTg Mlkl*$^{-/-}$ *Ikk2*$^{M\text{-}KO}$ with H/E and toluidine blue staining (n=6-12) at the age of 10 weeks; Scale bar: 500 µm. Data are presented as the mean ± SEM *P < 0.05 and ****P<0.0001 by one-way ANOVA (Bonferroni correction).
