# Supplementary material for: Cyld restrains the hyperactivation of synovial fibroblasts in inflammatory arthritis by regulating the TAK1/IKK2 signaling axis
Source: Cell Death Dis. 2024 Aug 9;15(8):584. doi: 10.1038/s41419-024-06966-2 (PMC11316070; doi:10.1038/s41419-024-06966-2)
Supplement: Supplementary file 2 — Supplementary Tables [file 41419_2024_6966_MOESM2_ESM.pdf]

Supplementary Table 1. Antibodies

Antibody	Manufacturer	Cat No	Dilution
Cyld	SantaCruz Biotechnology	sc-74434	1:400
Ikk2	Cell Signaling	8943	1:1000
p-Ikk1/2	Cell Signaling	2697	1:1000
JNK2	SantaCruz Biotechnology	sc-7345	1:400
p-JNK1/2	Cell Signaling	4668	1:1000
p-IkB	Cell Signaling	2859	1:1000
IkB	SantaCruz Biotechnology & Cell Signaling	SC143 & 9242S	1:1000
p-p38	Cell Signaling	9215	1:1000
p38	SantaCruz Biotechnology	sc-7972	1:500
p-ERK1/2	SantaCruz Biotechnology	sc-7383	1:1000
ERK	SantaCruz Biotechnology	sc-154	1:500
TAK1	Cell Signaling & SantaCruz Biotechnology	5206 & sc-7162	1:1000
p-TAK1	Cell Signaling	4508	1:500
TAB1	SantaCruz Biotechnology	sc-166138	1:500
K63 ubiquitin	Cell Signaling	5621	1:1000
Ubiquitin	SantaCruz Biotechnology	sc-166553	1:1000
Lamin B	SantaCruz Biotechnology	sc-6216	1:500
β Tubulin	Cell Signaling & SantaCruz Biotechnology	2146 & sc-58884	1:1000
β Actin	SantaCruz Biotechnology	sc-47778	1:1000
HRP Goat Anti-Rabbit IgG (H+L)	Biotium	20402	1:2000
HRP Goat Anti-Mouse IgG (H+L)	Biotium	20401	1:2000
**FACS Antibodies**			
CD11b (APC)	Biolegend	101211	1:100
CD11c (PE/Dazzle 594)	Biolegend	117347	1:100
CD45 (APC-Cy7)	Biolegend	103116	1:100 (1:400, SF culture)
NK1.1 (PE)	Biolegend	108707	1:100
Ly-6G (PE-Cy7)	Biolegend	127617	1:100
Ly-6C (FITC)		128005	1:100
Pdpn (PE-Cy7)	Biolegend	127411	1:100
Thy1.2 (Alexa Fluor 647 & 488 for SF culture)	Biolegend	105318 or 105315	1:100 (1:300, SF culture)
CD31 (APC/Fire™ 750)	Biolegend	102433	1:100
Ter119 (APC/Fire™ 750)	Biolegend	116249	1:100
VCAM-1 (Alexa Fluor 647)	Biolegend	105712	1:400
ICAM-1 (PE)	BD Pharmigen	553253	1:400

Supplementary Table 2. Oligos

IL-1b	F: CTGAAGCAGCTATGGCAACTG<br>R: TTTCAGCTCATATGGGTCCGA
IL-6	F: CTTCTTGGGACTGATGCTGGTGAC<br>R: TCCAGGTAGCTATGGTACTCCAGA
Mmp3	F: GTCTCCCTGCAACCGTGAA<br>R :CCACCCTTGAGTCAACACCT
Mmp9	F: CCTGTGTGTTCCCGTTCATCT<br>R: CGCTGGAATGATCTAAGCCCA
Mmp13	F: GATGACCTGTCTGAGGAAGACC<br>R: GCATTTCTCGGAGCCTGTCAAC
Timp1	F: ACAAGTCCCAGAACCGCAGTA<br>R: GGACCTGATCCGTCCACAAAC
β2m	F: TTCTGGTGCTTGTCTCACTGA<br>R: CAGTATGTTCGGCTTCCCATTC
