# Supplementary figures and images for: Cyld restrains the hyperactivation of synovial fibroblasts in inflammatory arthritis by regulating the TAK1/IKK2 signaling axis
Source: Cell Death Dis. 2024 Aug 9;15(8):584. doi: 10.1038/s41419-024-06966-2 (PMC11316070; doi:10.1038/s41419-024-06966-2)

Figure 4

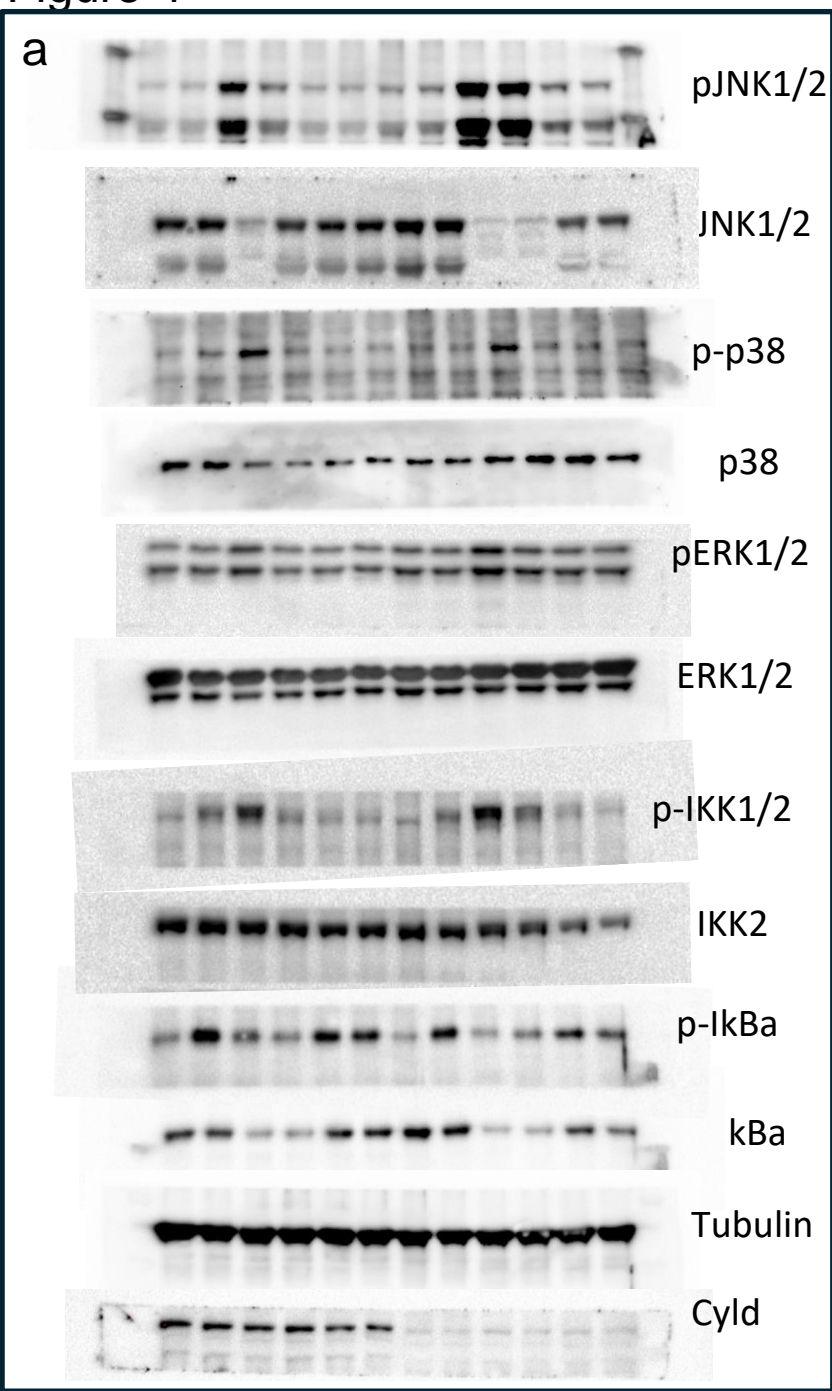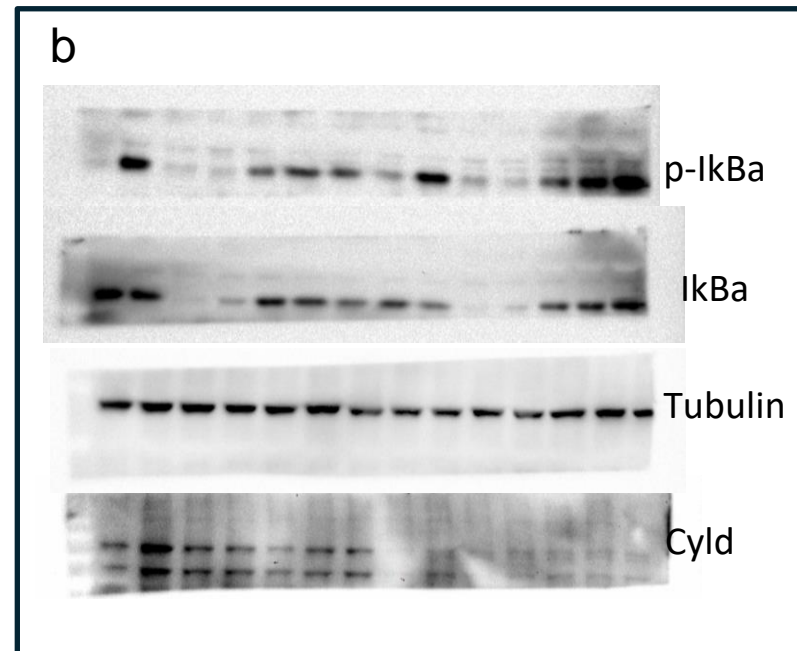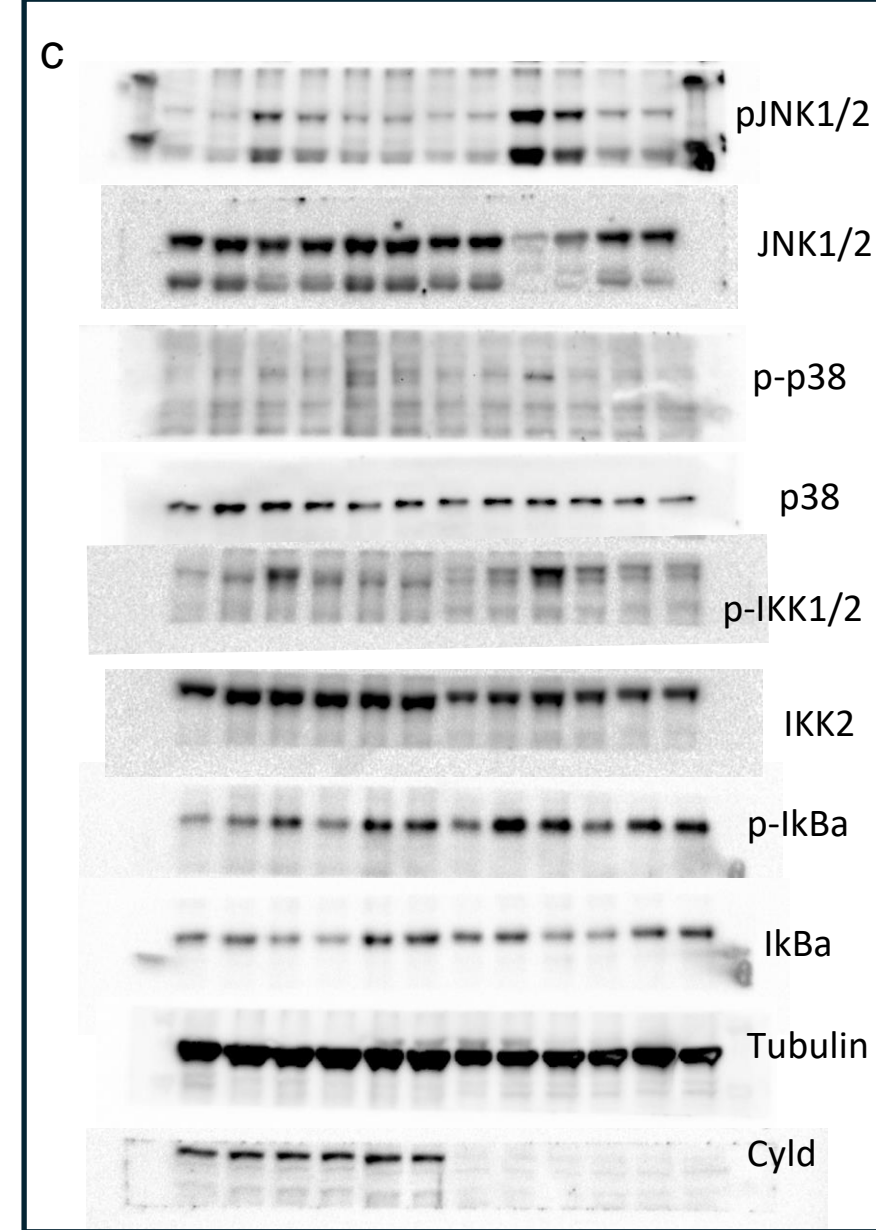

Figure 5

a

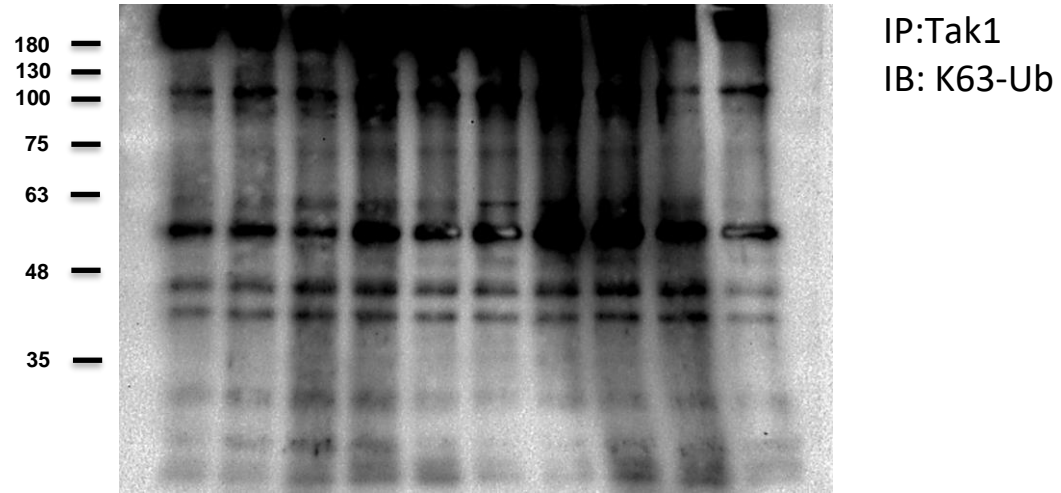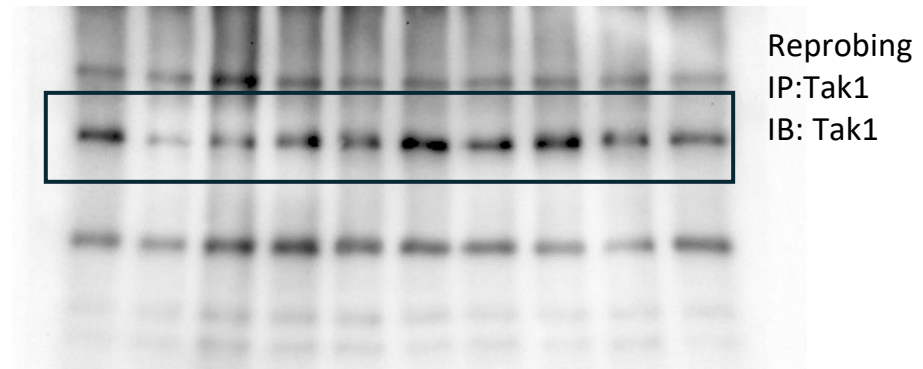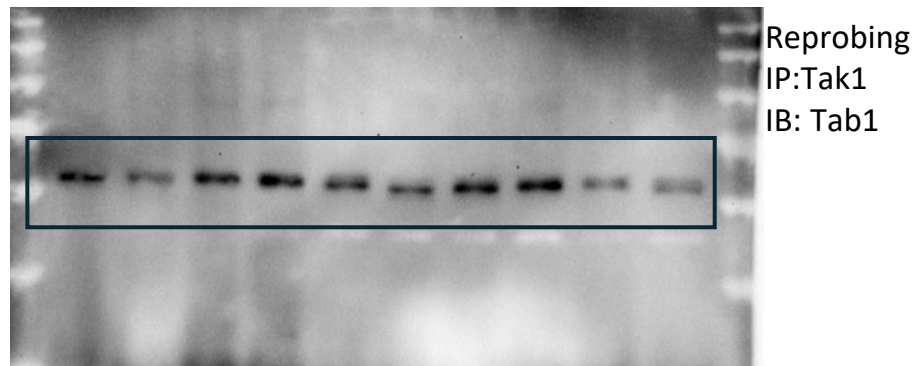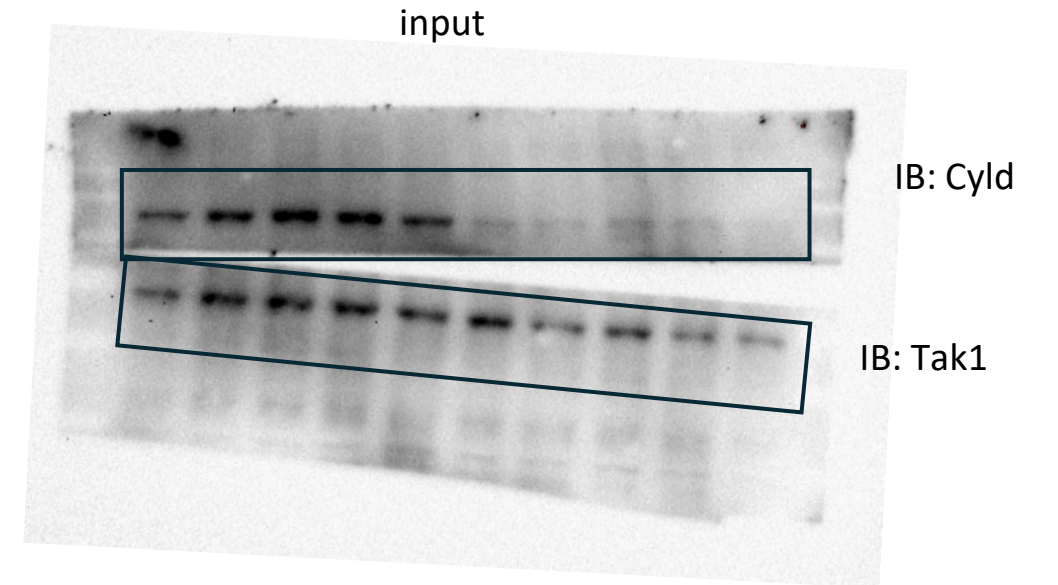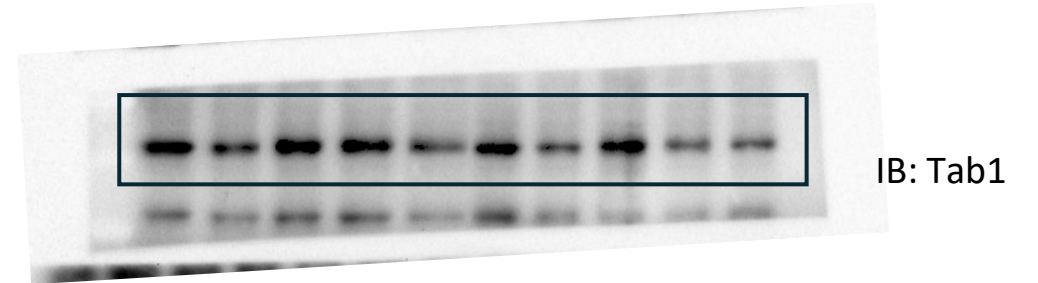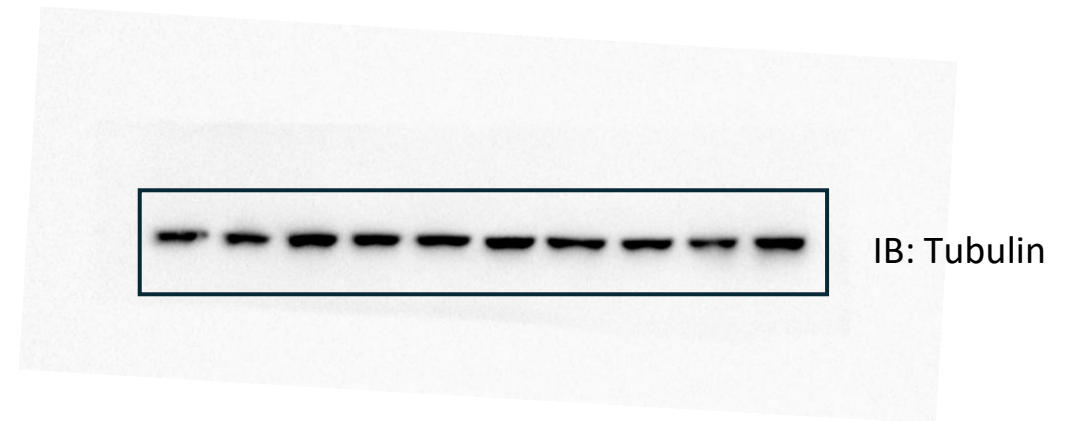

**b**

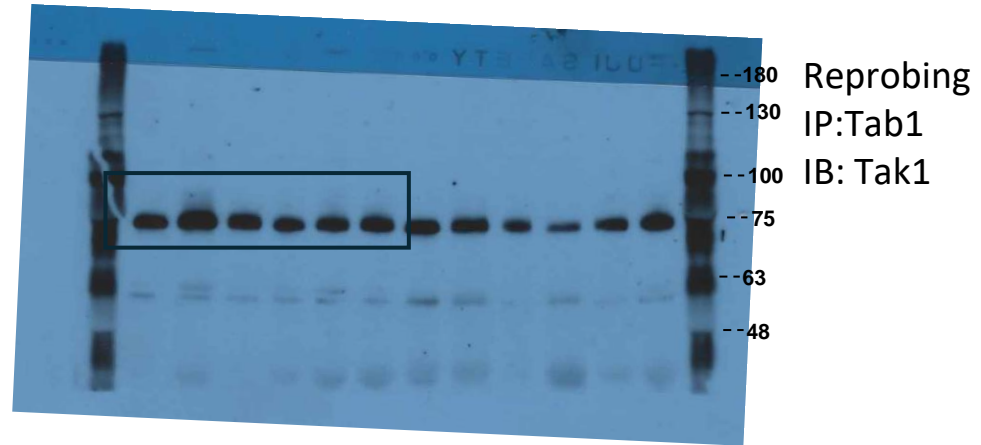

## Supplementary Figure 2

e

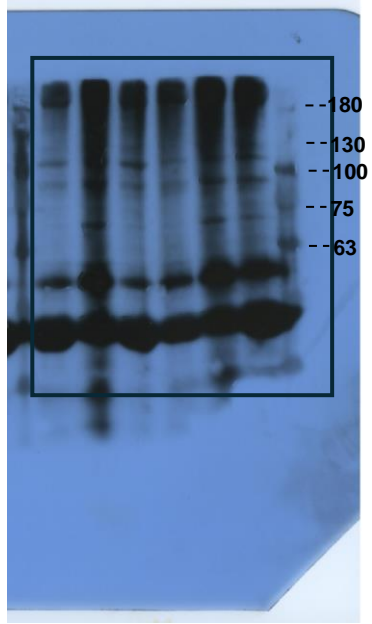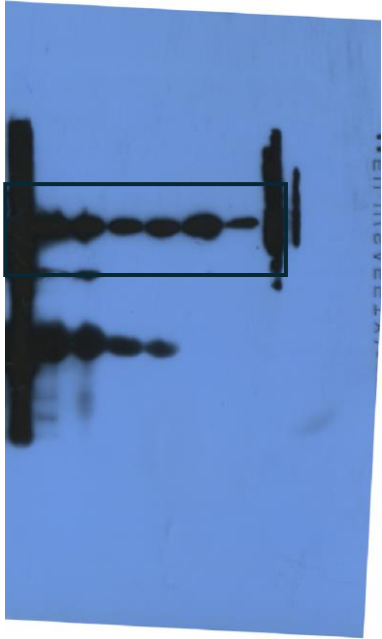

Supplement: Supplementary file 3 — Uncropped Immunoblots [file 41419_2024_6966_MOESM3_ESM.pdf]
